# Hydrometallurgical Process for Tantalum Recovery from Epoxy-Coated Solid Electrolyte Tantalum Capacitors

**DOI:** 10.3390/ma12081220

**Published:** 2019-04-14

**Authors:** Wei-Sheng Chen, Hsing-Jung Ho, Kuan-Yan Lin

**Affiliations:** 1 Department of Resources Engineering, National Cheng Kung University, No.1 University Road, Tainan City 701, Taiwan; kenchen@mail.ncku.edu.tw; 2 Department of Environmental Studies for Advanced Society, Graduate School of Environmental Studies, Tohoku University, Aoba-6-6 Aramaki, Aoba-ku, Sendai 980-8577, Miyagi, Japan

**Keywords:** tantalum capacitors, recycling, hydrometallurgy, pressure leaching, solvent extraction

## Abstract

Tantalum is a critical metal that is widely used in electronic products. The demand for tantalum is increasing, but the supply is limited. As tantalum waste products have increased in Taiwan in recent years, the treatment of spent tantalum capacitors has become necessary and important. The recycling of tantalum from tantalum capacitors will not only decrease pollution from waste, but will also conserve tantalum resources. The tantalum content in epoxy-coated solid electrolyte tantalum capacitors (EcSETCs) is over 40 wt.%. Here, we designed a recycling process that includes pre-treatment, leaching, and solvent extraction to recover tantalum. In the pre-treatment process, epoxy resin and wires were removed. During hydrometallurgical process, pressure leaching by hydrofluoric acid was used to leach tantalum and manganese from solid electrolyte tantalum capacitors (SETCs). During our testing of this proposed process, the acid concentration, reaction time, temperature, and solid–liquid ratio were examined for leaching. After the leaching process, Alamine 336 was used to extract tantalum from the leaching solution. The pH value, extractant concentration, extraction time, and aqueous–organic ratio were investigated. Then, tantalum was stripped using HNO_3_, and the HNO_3_ concentration, stripping time, and organic–aqueous ratio were analyzed in detail. Under optimal conditions, the recovery efficiency of tantalum reached over 98%, and a final product of tantalum pentoxide with 99.9% purity was obtained after chemical precipitation and calcination.

## 1. Introduction

Tantalum (Ta) is an important metal found in the Earth’s crust, having a high melting point (3017 °C), high corrosion resistance to acids at normal temperatures, and high conductivity of heat and electricity. Due to those unique properties, tantalum has become indispensable in current industries. Most tantalum production involves the manufacturing of capacitors (34%), which have higher performance than other types of capacitors. Tantalum is also used as specialty chemicals (11%), sputtering targets (13%), super alloys (23%), mill products (10%), and carbide products (9%) [1,2].

The Tantalum–Niobium International Study Center (TIC) indicates that the global production of tantalum was 2800 tons in 2014, and at that time, it was sourced from 1400 tons of tantalum mineral concentrate, 370 tons of tin slag, and 990 tons of scrap, respectively [3]. Due to recent developments, the recycling of tantalum has gradually begun to play an important role in the supplementation of tantalum reserves. Yen et al. noted that Taiwan imported 340 tons of tantalum products in 2013 and that the major use of tantalum in Taiwan was in capacitors, accounting for 61.6% of the total use at the time [4]. In 2016, the Customs Administration report indicated that Taiwan imported 354 tons of tantalum product. About 80% of the total imported tantalum products remained in Taiwan. The major use of tantalum, again, was in capacitors, accounting for 67%. However, it must be noted that these tantalum products will all eventually become tantalum waste. Because tantalum waste has dramatically increased in recent years, the tantalum recycling process must be studied. Additionally, tantalum ore always contains a considerable amount of niobium, and a lot of energy is required to remove niobium during the tantalum refining process [5]. The problem is that niobium and tantalum have similar chemical characteristics, which makes refinement difficult. However, the tantalum used in capacitors is free of niobium. Hence, studying the recovery of tantalum from capacitors is not only beneficial for waste management, but it also avoids the disadvantages of the typical tantalum recovery process.

Epoxy-coated solid electrolyte tantalum capacitors (EcSETCs) consist of epoxy resin, wires, and electrodes. Epoxy resin is made of halogenated compounds to which silica is added to enhance its thermal durability. The wires are made of iron and nickel. Electrodes, including cathodes, anodes, and dielectrics, are made of tantalum powder and other elements. Anodes and dielectrics are made of tantalum, and cathodes are made of manganese dioxide, silver paste, and graphite, which are daubed on the surface of the electrode as a cathode layer.

EcSETCs are sealed with epoxy resin, which reduces the efficiency of tantalum recovery; hence, the epoxy resin must be removed before any hydrometallurgical processes may be conducted. Many methods can decompose epoxy resin, such as combustion [6], solubilization [7], and super critical water treatment [8]. Those methods have been proposed in previous studies, and steam gasification [9] has been investigated to prevent the generation of harmful halogen compounds in combustion. In the hydrometallurgical process, tantalum capacitors should be dissolved by leaching before purification. In the traditional leaching process, leaching agents, such as, H_2_SO_4_ [10,11], hydrofluoric acid (HF) [12,13], or H_2_SO_4_ mixed with HF [14], at normal temperatures have been investigated but have been found to achieve only low leaching efficiency. In addition, Wang et al. [15] applied alkali leaching with roast-water, and Rodriguez et al. [16] applied pressure leaching using hydrofluoric acid. Both methods reached high leaching efficiency. Hence, in this study, the leaching process will be improved by combining the advantages of both methods. With respect to solvent extraction, several extractants, such as MIBK [17,18,19,20,21,22,23,24,25], TBP [19,26,27], CHO [19,22], OCL [28], and Alamine 336 [27,28,29,30,31,32] have been tested. Previous studies have indicated that Alamine 336 is a promising extractant for purifying tantalum in low HF concentrations [32]. Hussaini et al. indicated that tantalum could be stripped using ammonium or nitric acid in Alamine 336 [31].

Due to the increasing demand for tantalum, the recovery of tantalum from EcSETCs provides new opportunities and alternatives for conservation and waste disposal in Taiwan. As discussed previously, many tantalum capacitors have remained in Taiwan, whereas there are few tantalum reserves. Hence, in this study, we designed a promising recycling process to treat tantalum waste and recover tantalum to obtain a secondary source of this important resource. In our proposed method, epoxy resin and wire are removed by pre-treatment before conducting a hydrometallurgical process. After pre-treatment, EcSETCs without epoxy resin, called solid electrolyte tantalum capacitors (SETCs), are obtained. Then, the SETCs are leached by acid via a leaching process. Subsequently, separation and purification processes are conducted. Here, parameters such as pH value, extractant concentration, extraction time and aqueous-organic ratio were investigated to optimize the extraction of tantalum during this process. Furthermore, during the stripping process, parameters such as concentration, reaction time, and organic–aqueous ratio were studied to achieve high stripping efficiency. After chemical precipitation and calcination, tantalum pentoxide is obtained as the final product of our proposed method. The optimized conditions in this study can be used to further develop the tantalum recovery process in future work.

## 2. Materials and Methods

### 2.1. Materials

The tantalum capacitors used in the experiment were EcSETCs from a Taiwanese manufacturer, with 220 μF/10 V. The EcSETCs were divided into three main components: wires, epoxy resin, and electrodes. The relative mass ratio of each component of the EcSETCs is shown in Table 1, and the concentration of elements in the EcSETCs is shown in Table 2. As shown in Table 1, the EcSETCs had 54.8 wt.% electrodes, 38.2 wt.% epoxy resin, and 7 wt.% wires. Table 2 shows the results of a chemical analysis of EcSETCs by inductively coupled plasma optical emission spectrometry (ICP-OES, Varian, Vista-MPX). The dominant component of the EcSETCs was tantalum, which was over 40 wt.%, and the other components included 6–8 wt.% manganese, 6–7% silicon, 3% iron, and 4% nickel.

### 2.2. Methods

The epoxy resin and wires had to be removed from the EcSETCs to raise the leaching efficiency. Hence, pre-treatment was necessary. Pyrolysis with nitrogen gas was shown to be capable of removing the epoxy resin from the EcSETCs, and it also prevented the generation of halogen gas. Thermogravimetric analysis (TGA, Perkin Elmer, Pyris Diamond TG/DTA) was used in the pre-treatment process to determine the temperature for pyrolysis. Then, grinding and washing with water was conducted to flush out the epoxy resin, which mainly contained silicon oxide. After the washing process, crushing with a crusher (Yuanjiou, YJ-T5520S) was conducted to enhance the efficiency of the leaching process due to the resulting increased contact surface. Finally, magnetic separation was used to remove iron and nickel from the wires. After the pre-treatment process, the resulting SETCs without epoxy resin were analyzed by X-ray diffraction (XRD; DX-2700, Dangdong, Liaoning, China), scanning electron microscopy (SEM; S-3000N, Hitachi, Tokyo, Japan), and energy dispersive X-ray spectrometry (EDS; XFlash6110, Bruker, Billerica, MA, USA).

During the leaching process, the SETCs were dissolved using inorganic acid. Different inorganic acids, such as HF (UniRrgion Bio-tech, 49%), nitrate acid (HNO_3_ Sigma-Aldrich HNO_3_, ≥65%), hydrochloric acid (HCl Sigma-Aldrich 37%), and sulfuric acid (H_2_SO_4_ Sigma-Aldrich 98%), were compared to select the best leaching agent. Parameters such as leaching with or without vapor pressure, acid concentration, temperature, reaction time, and solid–liquid ratio were investigated. The concentration was set from 1% to 49% *v*/*v* with vapor pressure at 23 atm and normal vapor pressure. The effect of concentration from 1% to 15% *v*/*v*, temperature from 100 to 220 °C, reaction time from 0.5 to 3 h, and a liquid–solid ratio from 5 to 100 mL/g were tested to enhance the leaching efficiency.

**Figure 1:**
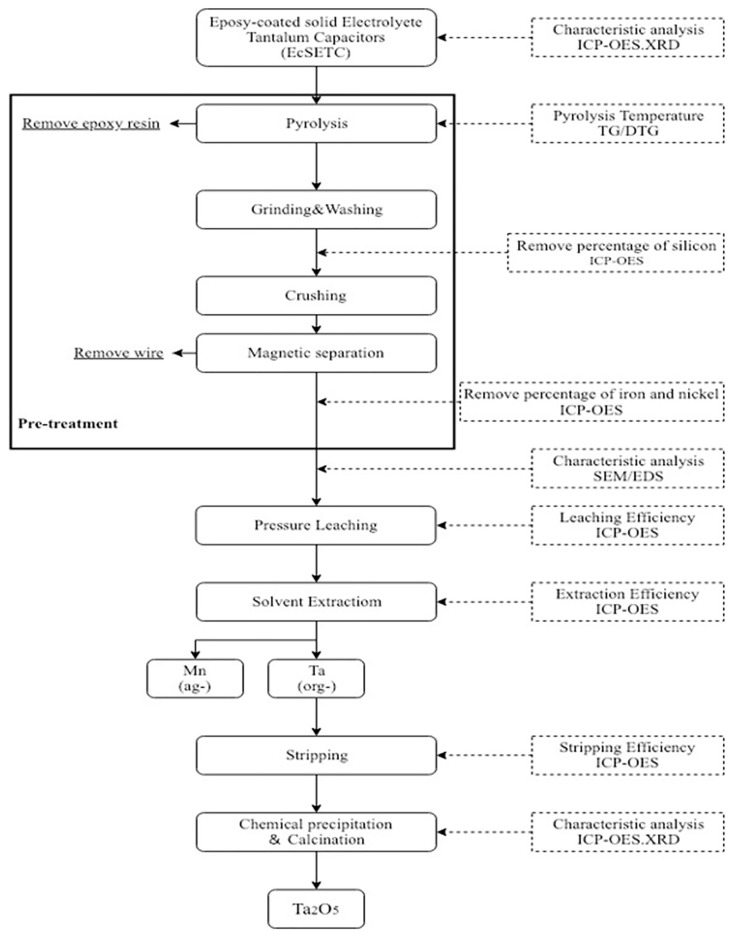
The proposed tantalum recovery process from EcSETCs. ICP-OES: inductively coupled plasma optical emission spectrometry; SEM: scanning electron microscopy; EDS: energy dispersive X-ray spectrometry; TG/DTG: Thermogravimetric analysis.

After the leaching process, solvent extraction was used to separate tantalum and other elements to increase the tantalum purity. TBP, Alamine 336, and MIBK diluted in kerosene were used as extractants for tantalum, and their extraction efficiency was analyzed. Then, parameters such as pH values from 1 to 5 in aqueous media, extractant concentration from 0.005 to 0.1 mol/L, aqueous–organic ratio from 0.5 to 9, and reaction time from 0.5 to 5 min were investigated in detail. Next, the organic phase with tantalum was stripped using HNO_3_. Parameters such as concentration from 0.5 to 3 mol/L, organic–aqueous ratio from 1 to 7, and stripping time from 0.5 to 5 min were investigated. Finally, after chemical precipitation and a calcination process, tantalum pentoxide was obtained as the final product. Figure 1 shows a flow chart of the proposed tantalum recovery process from EcSETCs.

## 3. Results and Discussion

### 3.1. Pretreatment

The EcSETCs were composed of epoxy resin, wires, and electrodes. Epoxy resin and wires connected with electrodes would decrease the leaching efficiency. Th epoxy resin was made of halogenated compounds and silicon oxide; on the other hand, the wire was made of iron and nickel. These elements would have also affected the purity of the final product. Thus, we designed a pre-treatment process to enhance the leaching efficiency and the purity of the final product.

**Figure 2:**
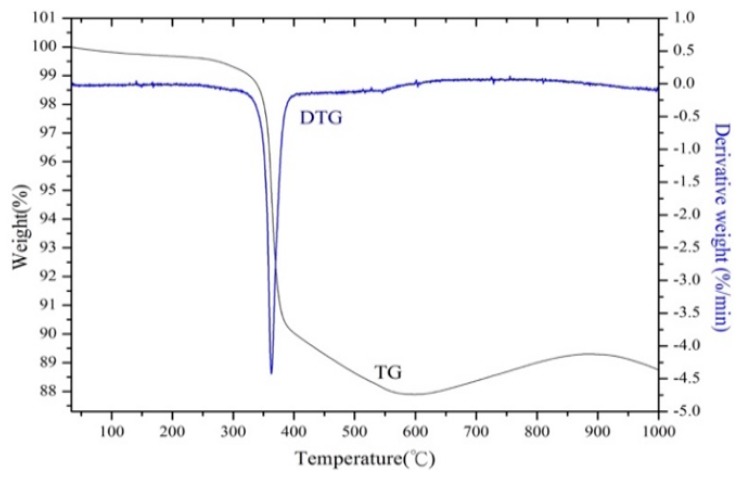
TG/DTG patterns of the EcSETCs with N_2_.

**Figure 3:**
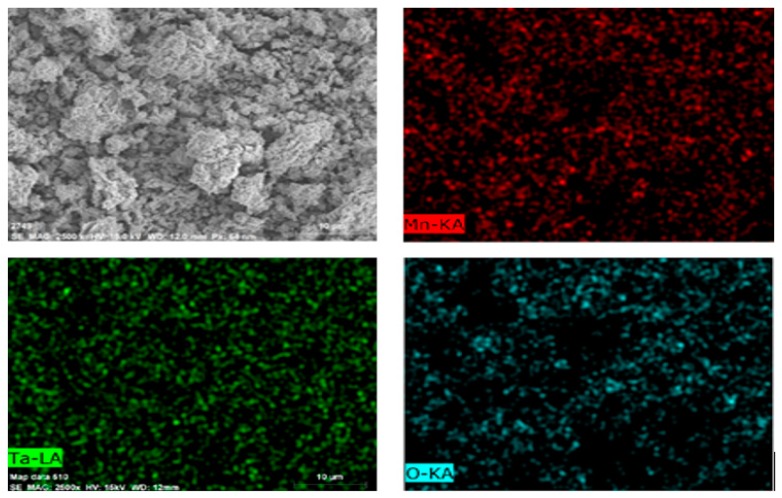
SEM-EDS analysis patterns of the solid electrolyte tantalum capacitors (SETCs) after pre-treatment.

The TG/DTG patterns are shown in Figure 2, which demonstrate that the epoxy resin began decomposing at 350 °C. By the time the temperature reached 600 °C, the EcSETCs had lost most of their mass because the epoxy resin had decomposed completely. According to results of the TG/DTG analysis, 600 °C for 10 min was selected as the optimal pyrolysis condition in the experiment. Grinding and washing with water was conducted after the pyrolysis process. Table 3 shows that silica could be removed by the pyrolysis process. Then, crushing was conducted before magnetic separation to ensure that tantalum and the other metals would be distributed uniformly. Table 4 shows that iron and nickel from the wires could be removed via magnetic separation. After subsequent pre-treatment processes, silicon, nickel, and iron were removed. Figure 3 shows SEM and EDS analysis patterns of the SETCs. As the results show, tantalum and manganese were distributed uniformly after the pre-treatment. In other words, it would have been hard to recover tantalum from the SETCs directly. However, here, the hydrometallurgical process successfully separated and recovered tantalum and manganese.

### 3.2. Leaching

The SETC powders were leached using four kinds of inorganic acids after pre-treatment. Table 5 shows that HF had the highest leaching efficiency with respect to tantalum of the four inorganic acids, because tantalum could be treated with hydrofluoric acid to produce water-soluble hydrogen fluorides. Therefore, HF was chosen as the leaching agent to leach tantalum from the SETC powders in the experiment.

**Figure 4:**
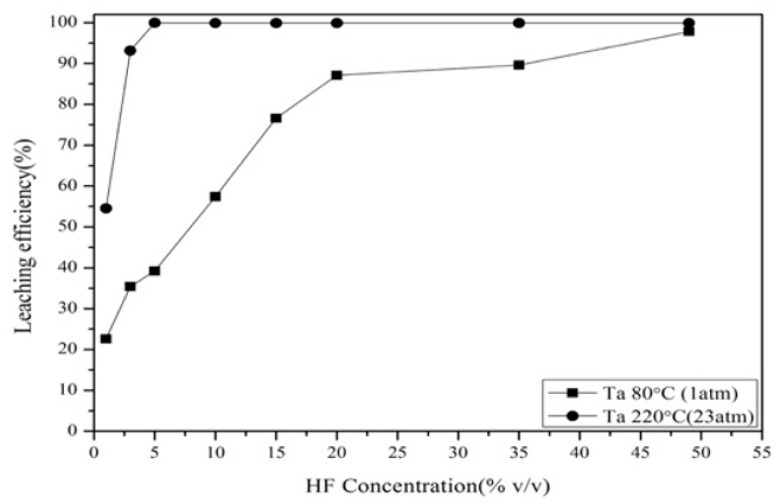
Effect of vapor pressure and normal pressure on tantalum leaching efficiency. Liquid–solid ratio: 100 mL per gram; reaction time: 3 h.

The effect of vapor pressure on the leaching of Ta was investigated. Figure 4 demonstrates that 5% *v*/*v* of HF was enough to leach Ta with 23 atm vapor pressure; on the other hand, leaching with normal pressure required 49% *v*/*v* of HF. According to the results, pressure leaching was used to leach Ta in this study. During the pressure leaching test, parameters such as hydrofluoric acid concentration, temperature, reaction time, and solid-to-liquid ratio were investigated.

#### 3.2.1. Effect of Hydrofluoric Acid Concentration

**Figure 5:**
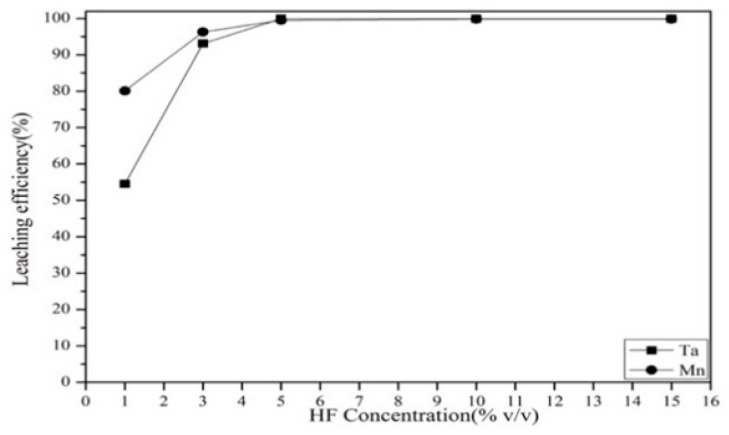
Effect of hydrofluoric acid concentration on the leaching efficiency. Temperature: 220 °C (23 atm). Liquid–solid ratio: 100 mL per gram. Reaction time: 3 h.

The effect of hydrofluoric acid concentration on the leaching of Ta and Mn was investigated from 1% to 15% *v*/*v* at 220 °C. The results are shown in Figure 5. The leaching efficiency of Ta and Mn increased as the HF concentration increased when HF concentration varied from 1% to 5% *v*/*v*. This may have been because an increased amount of F^−^ and H^+^ ions were available to react with the SETCs to form soluble complexes, which could be dissolved in water. The highest leaching efficiency was achieved when the HF concentration reached 5% *v*/*v*. Hence, 5% *v*/*v* of HF was chosen as the optimal concentration in the experiment.

#### 3.2.2. Effect of Temperature and Reaction Time

**Figure 6:**
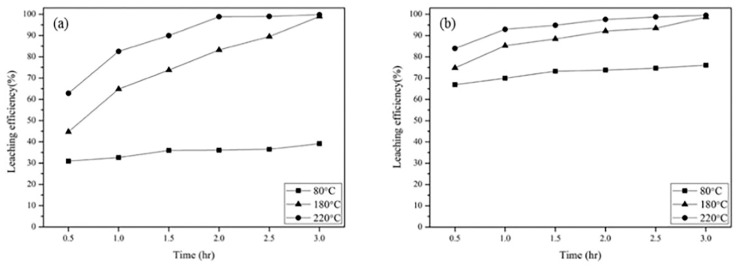
(**a**) Effect of the reaction time at different temperatures on Ta leaching efficiency. (**b**) Effect of the reaction time at different temperatures on Mn leaching efficiency. Concentration: 5 % *v*/*v*. Liquid–solid ratio: 100 mL per gram.

The effect of the reaction time on the leaching efficiency of Ta and Mn at different temperatures corresponding to different vapor pressures was studied. Figure 6a shows the effect of the reaction time at different temperatures on the Ta leaching efficiency. Figure 6b shows the effect of the reaction time at different temperatures on the Mn leaching efficiency. The results show that the Ta and Mn leaching efficiencies at 220 °C were faster than those at 180 °C. Ta and Mn required 3 h to reach the highest leaching efficiency in 180 °C but only 2 h in 220 °C. Nevertheless, both temperatures could achieve almost the same leaching efficiency. Thus, we could choose the appropriate parameters according to different process requirements and different demands of energy consumption, process cost, and leaching rate. In this study, we chose 180 °C for 3 h as the optimal temperature and reaction time.

#### 3.2.3. Effect of Liquid-to-Solid Ratio

**Figure 7:**
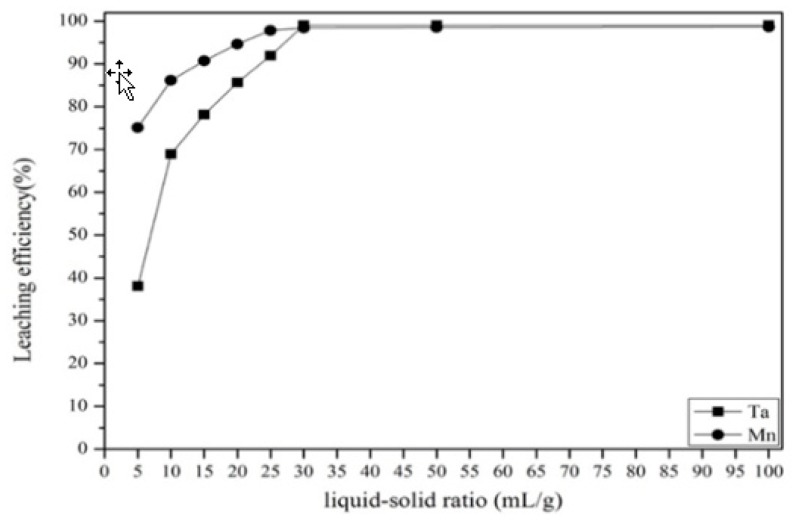
Effect of the liquid–solid ratio on the leaching efficiency. Reaction time: 3 h. Concentration: 5% *v*/*v*. Temperature: 180 °C.

The effect of the liquid-to-solid ratio from 5 to 100 mL/g was studied. Figure 7 shows that the leaching efficiency was increased when the liquid–solid ratio increased from 5 to 30 mL/g. This was because, when the liquid–solid ratio was low, there was insufficient acid to react in this process. Hence, the optimal liquid–solid ratio was identified to be 30 mL/g.

Based on the results, the optimal conditions for enhancing leaching efficiency were determined to be 5% *v*/*v* of HF in a liquid–solid ratio of 30 mL/g at 180 °C for 3 h. With the optimal conditions, the leaching efficiency of Ta and Mn could reach 99%. Moreover, compared with previous research [16], with the same concentration of tantalum-rich powder, the HF consumption in this study was reduced by almost 70%, showing increased efficiency. Moreover, the leaching efficiency achieved in this study was almost 19% higher than those achieved in previous studies. The reason for this was attributed to the fact that most of the impurities in the samples were removed during the pre-treatment process.

### 3.3. Solvent Extraction and Stripping

After the leaching process, tantalum and manganese were dissolved in hydrofluoric acid. In order to separate tantalum and manganese, a solvent extraction process was conducted. In the solvent extraction experiment, the concentrations of Ta and Mn were diluted to 1000 mg/L and 150 mg/L, respectively. Here, hydrofluoric acid was used as the leaching agent and H_2_SO_4_ was used to adjust the pH value. A comparison of the extractants, the effect of the pH value, the extractant concentration, the aqueous-to-organic ratio, and the extraction time were investigated.

#### 3.3.1. Comparison of Extractants and Effect of pH Value

**Figure 8:**
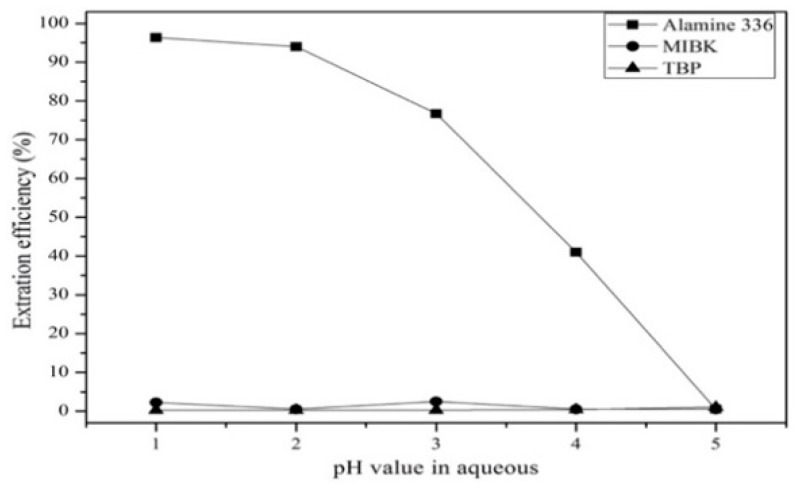
Effect of different extractants on the tantalum extraction efficiency. Concentration: 0.01 mol/L. Extraction time: 5 min. A/O = 1.

**Figure 9:**
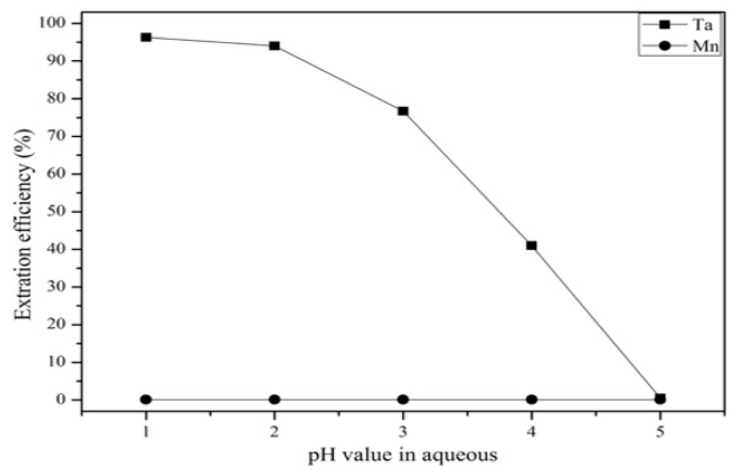
Effect of different pH values on the extraction efficiency. Concentration: 0.01 mol/L. Extraction time: 5 min. A/O = 1.

The effect of different extractants on the extraction efficiency were tested at pH values from 1 to 5. Figure 8 shows that Alamine 336 had the highest extraction efficiency with respect to tantalum of the three extractants. This was because Alamine 336 in HF-H_2_SO_4_ solution could extract tantalum at a low concentration. Figure 9 shows that the extraction efficiency of Ta decreased as the pH value increased. On the other hand, Mn was not extracted at any pH value. Hence, Alamine 336 and pH 1 were chosen as the extractant and optimal pH value in the solvent extraction experiment.

#### 3.3.2. Effect of Extractant Concentration, Aqueous-to-Organic Ratio, and Extraction Time

**Figure 10:**
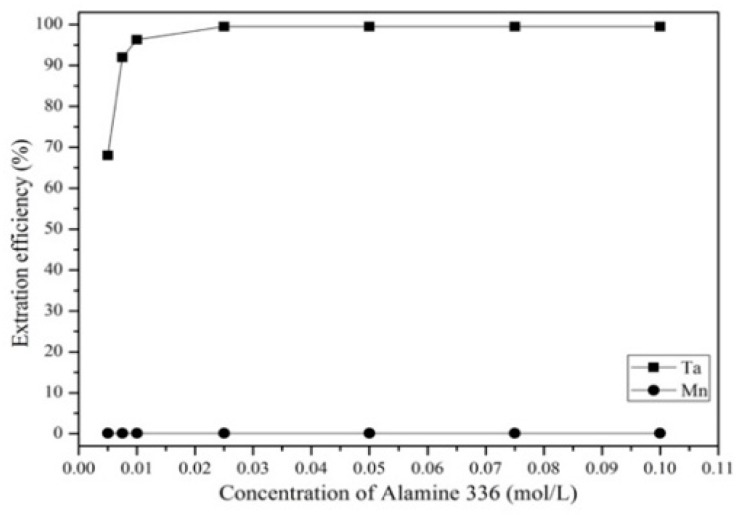
Effect of different concentrations of Alamine 336 on the extraction efficiency. pH = 1. Extraction time: 5 min. A/O = 1.

**Figure 11:**
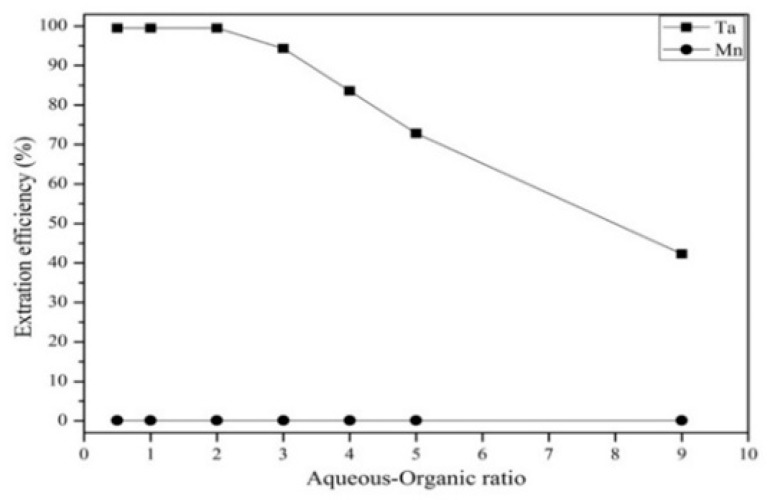
Effect of different aqueous–organic ratios on the extraction efficiency. pH 1. Extraction time: 5 min. Concentration: 0.025 mol/L.

**Figure 12:**
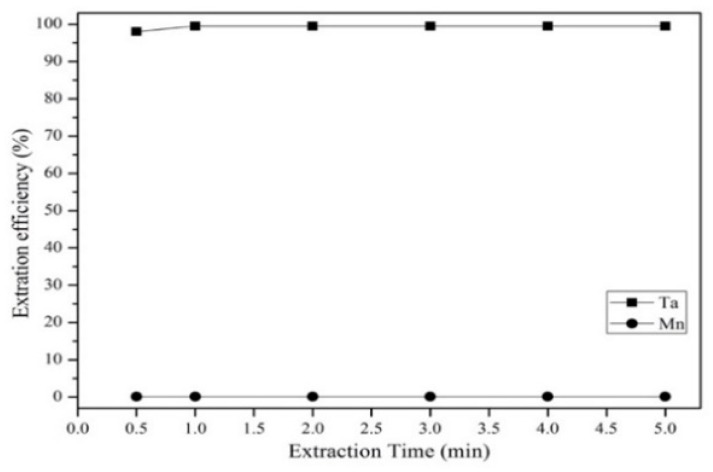
Effect of extraction time on extraction efficiency. pH 1. A/O = 2. Concentration: 0.025 M.

Figure 10 shows that the extraction efficiency of Ta increased as the Alamine 336 concentration increased from 0.005 to 0.025 mg/L. The extraction efficiency of Ta was highest at a concentration of 0.025 mg/L, which was chosen to be the optimal extractant concentration in the experiment. Figure 11 shows that the Ta extraction efficiency decreased as the aqueous-to-organic ratio increased from 2 to 9. Hence, we chose an aqueous-to-organic ratio of 2 as the optimal aqueous-to-organic ratio in the experiment. Figure 12 shows the effect of extraction time from 0.5 to 5 min. The results show that the extraction efficiency of Ta increased from 0.5 to1 min and the reaction was balanced after 1 min. Hence, in order to avoid too much energy consumption, we chose 1 min as the optimal extraction time.

Thus, the extraction efficiency of Ta was 99.5% and the extraction efficiency of Mn was 0.1% in the optimal conditions of 0.025 mol/L Alamine 336 at pH 1, for 1 min, and in an aqueous-to-organic ratio of 2. In solvent extraction process, the separation effect between Ta and Mn was demonstrated successfully.

#### 3.3.3. Effect of Stripping Efficiency on Agent Concentration, Organic-to-Aqueous Ratio and Stripping Time

**Figure 13:**
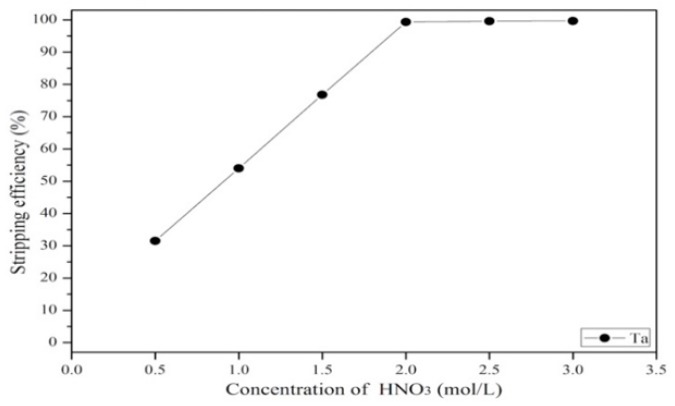
Effect of different concentrations of HNO_3_ on the stripping efficiency. Stripping time: 10 min. O/A = 1.

**Figure 14:**
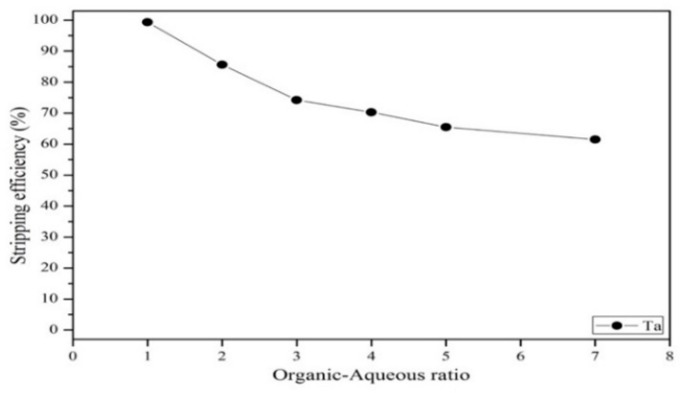
Effect of different organic–aqueous ratios on stripping efficiency. Stripping time: 10 min. Concentration: 2 mol/L HNO_3_.

**Figure 15:**
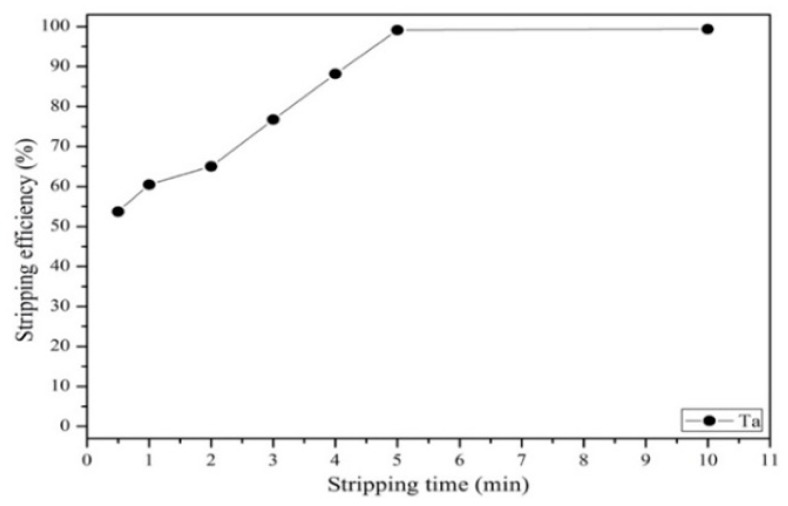
Effect of different stripping times on stripping efficiency. O/A = 1. Concentration: 2 mol/L HNO_3_.

After extraction, tantalum was stripped back to the aqueous phase from the organic phase via exposure to HNO_3_. Next, the concentration, stripping time, and organic–aqueous ratio were investigated. Figure 13 shows that the effect of different concentrations of HNO_3_ on the stripping efficiency. As the results show, the stripping efficiency of tantalum increased as the HNO_3_ concentration increased from 0.5 to 2 mol/L, and no increase in efficiency was observed when the concentration was higher than 2 mol/L. Hence, the optimal HNO_3_ concentration was 2 mol/L. Figure 14 shows that the stripping efficiency of tantalum decreased as the organic–aqueous ratio increased from 1 to 7. As the results show, an organic–aqueous ratio of 1 was optimal in the stripping process. Figure 15 shows that the stripping efficiency of tantalum increased as the stripping time increased from 0.5 to 5 min. After 5 min, the reaction was balanced. Finally, a stripping efficiency of tantalum of 99.4% was demonstrated under the optimal stripping parameters: 2 mol/L HNO_3_ with an O/A ratio of 1 and a stripping time of 5 min.

### 3.4. Chemical Precipitation and Calcination

**Figure 16:**
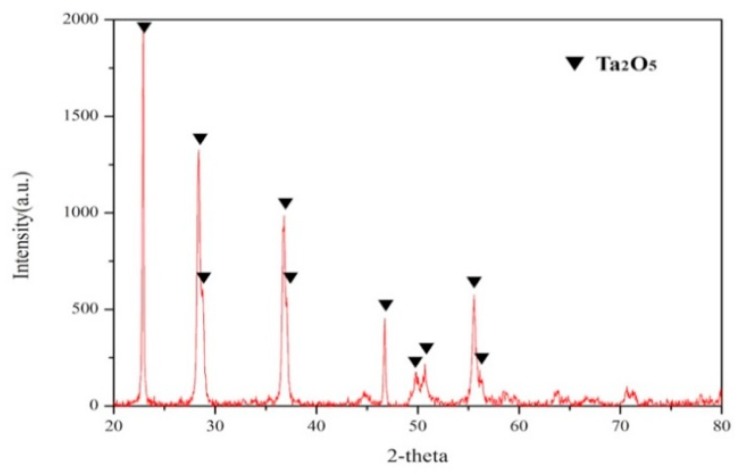
The XRD patterns for Ta_2_O_5_.

After the solvent extraction, stripping process, and selective precipitation, tantalum had already been separated from several impurities. Then, during the chemical precipitation process, tantalum was precipitated by ammonium hydroxide at pH value of 8 to obtain tantalum hydroxide. Thus, as a result of the calcination process, we obtained Ta_2_O_5_ as the final product. According to the final product analysis, the crystalline phase is demonstrated in Figure 16, and the purity of Ta_2_O_5_ was proven to be over 99.9% by ICP-OES.

## 4. Conclusions

As the demand for tantalum gradually increases, the issue of how to mitigate the exploitation of this natural resources has become very important. Hence, in this study, we designed and tested a recycling process for EcSETCs. The raw materials in this study were EcSETCs, which contained over 40 wt.% of tantalum. According to this composition, the EcSETCs had great potential for being recycled, which could provide a plentiful secondary tantalum source. During this recycling process, the EcSETCs were subjected to a pre-treatment, pressure leaching, solvent extraction, stripping, chemical precipitation, and calcination. The composition of the EcSETCs was 54.8 wt.% electrode, 38.2 wt.% epoxy resin, and 7 wt.% wire. In the pre-treatment process, the epoxy resin was removed by pyrolysis at 600 °C for 10 min, and then, the resulting SETCs were subjected to grinding and washing by water. The wires, which were made of iron and nickel, were also removed by magnetic separation. The optimal pressure leaching conditions were determined to be 5% *v*/*v* of HF in a liquid-to-solid ratio of 30 mL/g at 180 °C for 3 h. With the optimal conditions, the leaching efficiency of Ta and Mn could achieve 99%. In the solvent extraction process, Ta and Mn were separated under 0.025 mol/L Alamine 336 at pH 1 for 1 min with an aqueous-to-organic ratio of 2. The extraction efficiency of Ta was 99.5%; however, the extraction efficiency of Mn was only 0.1%. In the stripping process, under the optimal stripping parameters—2 mol/L HNO_3_ at an organic–aqueous ratio of 1 with a stripping time of 5 min—the stripping efficiency of Ta was 99.4%. Then, after chemical precipitation and calcination, the final product of Ta_2_O_5_ was obtained; moreover, the purity of Ta_2_O_5_ was over 99.9%, and the recovery efficiency of tantalum reached over 98% as a result of our proposed recovery process. Thus, our proposed recycling process was proven to be successful and effective, and the detailed reaction mechanism and optimization of this process will be discussed in future work.

## Figures and Tables

**Table 1:** Mass ratio of each component in the epoxy-coated solid electrolyte tantalum capacitors (EcSETC) (wt.%).

Tantalum Electrode	Epoxy Resin	Wire
54.8%	38.2%	7%

**Table 2:** Concentration of elements in the EcSETCs.

Element	Ta	Si	Mn	Fe	Ni
Wt.%	40–42%	6–7%	6–8%	3%	4%

**Table 3:** Removed percentage of silicon in the sample after pyrolysis, grinding, and washing.

Table 3	Si	Ta
Before	400 mg/kg	4500 mg/kg
After	0.2 mg/kg	4500 mg/kg
Remove percentage	99.9%	0%

**Table 4:** Remove percentage of nickel and iron after magnetic separation.

Table 4	Ni	Fe	Ta
Mag.	99.5%	99.5%	0%
Non-Mag.	0.5%	0.5%	100%
Removed percentage	99.5%	99.5%	0%

**Table 5:** Leaching efficiency with respect to tantalum of the different inorganic acids.

Table 5	Leaching Efficiency of Ta (%)
Hydrofluoric acid (HF)	17.57%
Hydrochloric acid (HCl)	0.13%
Sulfuric acid (H_2_SO_4_)	0.01%
Nitric acid (HNO_3_)	0.15%

(Concentration: 1% *v*/*v*; reaction time: 3 h; temperature: 25 °C; liquid–solid ratio: 100 mL per gram.)
